# Effect of Nematodes-Bacteria Complex Metabolites on Cancer and Tumor Progression

**DOI:** 10.3390/biom15081165

**Published:** 2025-08-14

**Authors:** Aisa Bahar, Malihe Parsa Sefat, Meisam Khazaei, Hamed Tahmasebi, Valentyn Oksenych

**Affiliations:** 1 School of Medicine, Shahroud University of Medical Sciences, Shahroud 36147-73943, Iran; 2 Biochemistry Department, Faculty of Medicine, Iran University of Medical Sciences, Tehran 14496-1453, Iran; 3 Faculty of Medicine, University of Bergen, 5020 Bergen, Norway

**Keywords:** gastrointestinal helminths, gastrointestinal microbiota, cancer, gut microbiota, apoptosis

## Abstract

Helminths that inhabit the gastrointestinal (GI) tract represent some of the most significant infectious agents impacting health. The interaction between the human microbiota, GI helminths, and their host occurs through multiple complex pathways, altering the host’s immune system and the dynamics of the commensal gut microbiota (GM). These interactions also largely influence a balanced state of homeostasis and health promotion and robustly activate the immune system, facilitating tumor eradication and mitigating the challenges of drug resistance. Furthermore, incorporating microbial metabolites into radiotherapy and chemotherapy reduces the intense adverse effects of these treatments while enhancing their overall effectiveness. The interplay between GM and helminths, as well as their metabolites, significantly impacts the development, prognosis, and treatment of cancer. The interaction mechanisms between GI helminths and the GM are not fully elucidated. Thus, understanding a beneficial biological relationship can reveal hidden mechanisms for controlling and inhibiting cancer pathways in humans by providing insights into cellular processes and potential therapeutic targets. This knowledge can be applied to develop more effective cancer treatments. This review outlines the existing research on GM metabolites in cancer, intending to offer innovative pathways for future cancer treatment.

## 1. Introduction

A recent analysis of cancer statistics showed that close to 20 million new cancer diagnoses occurred worldwide in 2022 and that 9.7 million deaths were associated with cancer [1]. As a significant cause of death, cancer has become a leading contributor to human mortality rates. In recent years, groundbreaking methods have emerged for cancer prevention and therapy, including using antibody-drug conjugates alongside immunotherapy [2,3]. Despite the advancements these therapeutic methods offer for tumor intervention, their success is limited by the resistance of tumor cells to pharmacological agents and the complex tumor microenvironment (TME), which creates obstacles in effective tumor therapy. The uneven structure of blood vessel networks in the TME hinders the effective delivery of chemotherapy drugs [4,5].

Moreover, cells that suppress immune activity within the TME possess the ability to weaken immune responses, consequently diminishing the success of immunotherapy. Research into novel adjuvant medications is designed to augment the success of established therapies, minimize patient distress, and aim to achieve a cancer-free outcome. A recent study suggests that a lack of microbiota in the TME impairs monocyte production of type I interferon (IFN-I) [6,7]. IFN-I affects the growth and survival of cancer cells [8], and an important effect of the lack of IFN-I is macrophage polarization to M2 and immune evasion [9]. Consequently, an immunosuppressive environment forms, helping tumor cells escape recognition and elimination by immune defenses. Changes in the microbiome affect tumor growth, and restoring gut microbial balance through fecal microbiota transplantation (FMT) may offer a viable treatment option. Chronic inflammation triggered by the microbiota may lead to cancer progression. The metabolites produced by microbiota play a significant role in cancer and its prevention [10,11].

Metabolites from microbes could enhance current treatment methods as supplementary agents. Butyric acid, produced by gut bacteria, modulates T-cell receptor pathways to enhance the release of cytokines with cancer-fighting properties. Butyric acid significantly enhances the tumor-fighting abilities of immune cells, thereby increasing the efficacy of immunotherapy targeting programmed cell death protein 1 (PD-1). There exists a two-way relationship between microbial metabolites and the effectiveness of clinical therapies [12,13]. Through its metabolic activity, *Fusobacterium nucleatum* produces succinic acid, which diminishes the synthesis of IFN-β and limits the infiltration of CD8+ T-cells into the TME. Therefore, the immune system’s ability to combat the tumor is reduced [14]. Consequently, microbial metabolites can oppose tumor therapy, offering both advantageous and detrimental effects. Understanding the dual impact involves clarifying the precise mechanism by which it operates and considering the specific contexts that shape its effects. The therapeutic implications for tumors vary with the levels of short-chain fatty acids (SCFAs) present. High SCFA concentrations in non-alcoholic fatty liver disease may lead to hepatocellular carcinoma if they go beyond the host’s tolerance level. Normal levels of SCFAs significantly slow down colorectal cancer progression [15,16,17]. The relationship between microbial metabolites and tumors is not straightforward; their effects change with concentration. The impact of helminth-bacteria interactions on generating or reducing microbial metabolites is yet to be clear [18,19].

Helminths and microbiota influence each other evolutionarily. *Trichuris* genus parasites inhabit mammalian intestines, with eggs developing in soil but hatching in the cecum only with bacteria present. *Trichuris muris* cannot colonize germ-free mice, as egg hatching depends on interaction with bacterial fimbriae [20,21]. Worms need their host’s intestinal microbiota for full maturation; disrupting this acquisition hampers development. By altering the microbiota, worms may prevent other infections, influencing new *Trichuris* worms [21,22]. Laboratory mice returned outdoors show increased susceptibility to *T. muris* due to a diverse microbiota and an immune response favoring type 1 cytokines like IFNγ. The link between higher worm load and changes in microbiota or immune reactions is unclear, but it is not just due to egg hatching; worms have more biomass and thrive outdoors [23,24]. Early *Trichuris* infection shows a strong IFNγ presence, including Isg15 expression, as larvae form syncytial tunnels in epithelial layers. Worms might use the host’s IFNγ response to boost colonization and proliferation within the intestine [25,26].

**Figure 1:**
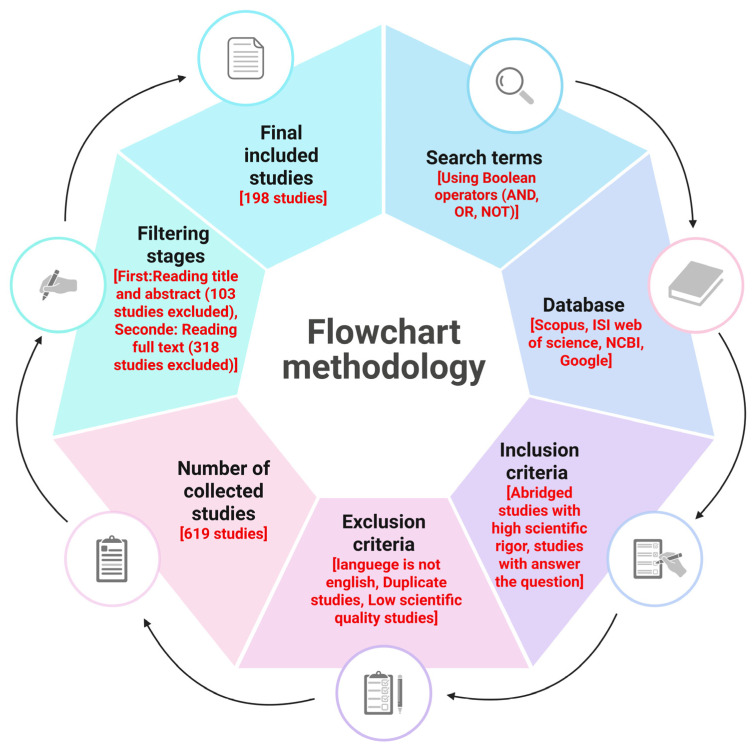
The flowchart outlines the essential steps in conducting a critical literature review, encompassing inclusion and exclusion criteria as well as filtering stages. It is important to note that scientific rigor within the inclusion criteria is assessed through the application of appropriate scientific methods. These methods ensure unbiased and robust design, methodology, analysis, interpretation, and reporting throughout the review process. This figure was generated using BioRender software version 04.

The interplay between helminths and bacteria may not always have negative consequences; it could aid in developing therapies for intestinal inflammation and related cancers. The immune response to helminth-bacterial infections varies with species and density, indicating a degree of tolerance. Helminths are ancestral microorganisms that have evolved alongside their hosts, competing with beneficial microbes and influencing gut immune regulation, especially early in life [22,27]. This review examines the relationship between intestinal parasites and microbiota in tumor progression, focusing on inflammation, necrosis, apoptosis, and the roles of their metabolites in advancing tumors. Additionally, we show in Figure 1 a flowchart methodology with inclusion and exclusion criteria of articles.

## 2. Role of Microbial Metabolites in Tumor Progression

The balance of intestinal flora is vital in controlling hemostatic processes within the intestines. Identifying the microbiota factors that influence these effects has not been achieved thus far. A microbial community known as microbiota consists of various microorganisms, including bacteria, archaea, viruses, and select unicellular eukaryotes, that thrive in specific environments; the aggregate genomic data of these microbes is collectively referred to as the microbiome [28,29].

### 2.1. Sources and Classification of Gut Microbial Metabolites

Gut microbes produce metabolites influenced by diet, including SCFAs, bile acids, phenolic compounds, vitamins, polyamines, tryptophan, and lipids. These play a crucial role in tumor development. The SCFAs are short-chain saturated fatty acids from fiber fermentation in the colon; they are absorbed in the large intestine [30]. Bacteria like *Fusobacterium rectum*, *Enterococcus faecalis,* and *Faecalibacterium prausnitzii* metabolize them [15]. Major SCFAs include acetate, propionate, and butyrate; butyrate is studied for its gut support and potential tumor inhibition to reduce colorectal cancer risk. The TA is an essential amino acid in protein-rich foods like poultry and soybeans; gut bacteria metabolize it into compounds that may affect the immune system [15,31].

In addition, the metabolic pathways characteristic of bacteria lead to the production of indole and its metabolites, which prominently include indole-3-aldehyde (IAld) and indole-3-acetic acid (IAA). These metabolites can activate the signaling pathways linked to the aryl pregnane X receptor (PXR) and the aryl hydrocarbon receptor (AHR), which can substantially influence the host’s immune response, thereby emphasizing their essential role in regulating immune activity [32,33]. The balance of the body’s immune responses, metabolic processes, and cellular reactions is significantly influenced by AHR and PXR pathways. These are crucial for maintaining homeostasis. While various gut bacteria contribute to these processes, research shows that the *Bacillus* genus plays a pivotal role due to its production of indole and its metabolites, essential for AHR and PXR functioning [34]. Enhancing *Bacillus* strains in our gut microbiota (GM) could optimize biological processes and improve health outcomes. Understanding these interactions highlights the importance of *Bacillus* in managing human health at a cellular level [35,36]. For example, *Bacillus sphaericus* produces indole from tryptophan, which can then be converted into IAld, oxidized to IAA, or transformed into tryptamine (TA) [34,37]. Inosine, produced from adenine deamination by gut bacteria, manages GM and maintains the intestinal barrier [38]. In addition, *Escherichia coli* metabolizes tryptophan into indole and pyruvate [39].

Foods like fish, eggs, and meat contain choline and carnitine, aiding trimethylamine oxide (TMAO) synthesis. Gut bacteria such as *Fusobacterium* convert these into trimethylamine (TMA), then into TMAO in the liver. The GM also converts primary bile acids into secondary ones that support fat metabolism. Polyphenols in fruits, vegetables, and herbs produce metabolites through gut bacteria that can affect cancer progression by boosting the immune system and reducing oxidative stress and inflammation [40,41,42].

#### 2.1.1. Short-Chain Fatty Acids

Acetate, propionate, and butyrate are the main SCFAs in the intestine, primarily from carbohydrate metabolism. Amino acid fermentation produces other acids like caproate and valerate. Acetate and propionate are found in both intestines, while butyrate is mainly in the cecum and colon [43]. Most gut species produce butyrate or propionate, not both. Propionate synthesis from dietary hexoses uses succinate with methylmalonyl-CoA decarboxylase (MMDA), crucial for producing propanoyl-CoA. A new pathway shows propane-1, 2-diol hydrolysis into propanol by GM like *Lachnospiraceae*, aiding deoxy sugar fermentation from glycans. Butyrate is synthesized through pathways using lysine, acetyl-CoA, glutarate, or 4-aminobutyrate substrates [44,45].

Ultimately, all these pathways converge at a critical juncture that involves either butyrate kinase (BUK) or butyryl-CoA transferase (BUT). Researchers recently found that the acetyl-CoA pathway represents the primary mechanism for butyrate production, making up about 80% of the pathways identified, while the lysine pathway holds the second position [46,47]. Bacteroidetes strains largely facilitate the production of butyrate through alternative metabolic pathways. The current comprehension of Bacteroidetes physiology casts doubt on the in vivo significance of these pathways, underscoring the necessity for further biochemical and physiological research, such as studies using isotopic tracers, to quantify the involvement of this phylum in the butyrate pool. The concurrent occurrence of lysine and acetyl-CoA pathways indicates a potential interaction with protein degradation processes, which enhances the system’s flexibility in adapting to a diet high in protein [45,48].

The SCFAs are crucial for colonic epithelial cells as an energy source and in maintaining the intestinal barrier by regulating tight junction proteins. Table 1 shows that certain SCFAs, like propionate and butyrate, reduce mucosal inflammation by interfering with NFκB-driven cytokine production, while acetate has a weaker effect. G-protein-coupled receptor 41 (GPR41) and GPR43 are key SCFA receptors, but other effects exist. GPR41 is found in various tissues and controls energy balance; mice without it show lower sensitivity to weight gain. The gut microbiota influences weight variation between germ-free and conventional mice. GPR43 is prominent in immune cells exposed to SCFAs; gene knockout studies reveal that SCFA-induced cytokine production depends on GPR43 in both cultured cells and living organisms [49,50,51]

#### 2.1.2. Aromatic Amino Acid (AAA) Derivatives

Important immunomodulatory metabolites produced by microbiota result from aromatic amino acids (AAA) degradation (Table 1). The kynurenine pathway is the primary method for tryptophan breakdown in humans and mice. Indoleamine 2,3-dioxygenase (IDO1) or tryptophan 2,3-dioxygenase (TDO) catalyzes the rate-limiting initial step, depending on the cell type. The aryl hydrocarbon receptor (AhR) has kynurenine as its natural ligand. The AhR primarily manages the transformation of xenobiotics when exposed to environmental chemicals. It plays a role in controlling immune responses and inflammation by influencing cytokine production and the development of Treg cells [52,53,54]. IDO1, found in immune cells in the intestine, regulates the tryptophan pool for microbiota metabolism, as kynurenine production competes with other pathways [55]. As shown in Table 1, the microbiota contributes significantly to the catabolism of tryptophan (TRP), resulting in the production of key metabolites such as TA, indole, and indole-3-acetamide (IAM). These compounds are synthesized through the enzymatic actions of tryptophanase A (TNA), tryptophan decarboxylase (TRD), and tryptophan 2-monooxygenase (TMO), respectively. Indole-containing compounds, such as IAld and IAA, can be derived from these products [53,56,57].

TRP reaction genes are extensively distributed across different GM lineages. They are found in both human and mouse genomes, with TNA being the only enzyme that is purely bacterial. Analyses of metabolomic profiles from intestinal, fecal, and blood samples of germ-free (GF), gnotobiotic, and conventionally raised (CONV-R) mice indicate that the GM plays a crucial role in the production of indole-containing metabolites and various amino acid derivatives, such as phenethylamine derived from phenylalanine and phenol originating from tyrosine [54]. TA, I3A, and IAld are AhR ligands akin to kynurenine and can alter the host’s immune responses. As a weak AhR agonist, indole diminishes TNFα production and NFκB activation in a dose-dependent manner, improves tight junction resistance in intestinal epithelial cells, and reduces inflammation markers, hinting that microbiota-derived TRP metabolites may affect host inflammatory pathways via different receptors [58,59].

In Table 1, the metabolism of AAAs highlights a new role of GM in influencing communication between the digestive and nervous systems. The gut’s enterochromaffin cells generate serotonin, an inhibitory neurotransmitter and hormone, through a process that converts TA via the sequential actions of the enzymes aromatic amino acid decarboxylase and tryptophan hydroxylase (TPH1). In GF mice, the serotonin levels in the serum are markedly lower; however, these levels return to normal when their gastrointestinal tract is populated with various Clostridium species, which also enhances the expression of TPH1 in the intestines [60,61].

#### 2.1.3. Bile Acids

Cholate (CA) and chenodeoxycholate (CDCA), the primary bile acids, are synthesized in the liver. They are conjugated with either glycine or taurine before entering the intestine, boosting their surfactant functionality. The role of microbial deconjugation in the metabolism of endogenous compounds is crucial for maintaining a balanced cycle of bile acids. Compounds derived from cholesterol support the absorption of dietary lipids and vitamins, and their synthesis rate is essential for maintaining cholesterol levels in the system [62]. In Table 1, deoxycholate (DCA) and lithocholate (LCA) are the primary secondary bile acids formed from the 7α-dehydroxylation of CA and CDCA, respectively. Several *Firmicutes* species, especially *Clostridium scindens*, possess the baiCD gene that facilitates bile acid dehydroxylation. However, the gene’s presence in colonic microbiota is not well understood. Conversely, bile salt hydrolases (BSH), which convert primary bile acids in the small intestine via deconjugation, have been thoroughly studied [63].

Through deconjugation and dehydroxylation, gut bile acids become more diverse. The farnesoid X receptor (FXR) is crucial in bile acid signaling, acting as a transcription factor in the liver and gastrointestinal tract [64]. Recent studies show that intestinal FXR negatively impacts liver bile acid production via fibroblast growth factor 15 (FGF15). Tauro-conjugated muricholic acid reduces FXR activity in mice. The GF mice have higher MCA and TβMCA levels than conventionally raised (CONV-R) mice, weakening FXR’s inhibition on bile acid production and increasing liver cholesterol metabolism through CYP7A1 [65]. *E. coli* with enhanced BSH variants affect BSH activity, bile composition, and host metabolism similarly in GF or CONV-R mice. Microbial metabolism of bile acids helps prevent harmful bacterial colonization in the gut; secondary bile acids adversely impact *Clostridium difficile* [66]. Studies using mouse models and clinical data found that *C. scindens* is linked to resistance against *C. difficile* infection by suppressing its growth via a bile salt-dependent mechanism. Differences between human and rodent bile acids pose challenges for using rodents as research models since human profiles consist only of CDCA and cholic acid (CA) [67,68].

In contrast, mice have a more diverse array of bile acids, comprising five distinct types: α-muricholic acid (α-MCA), CA, β-muricholic acid (β-MCA), CDCA, and ursodeoxycholic acid (UDCA). The regulatory processes governing bile acids in terms of cholesterol production and transformation exhibit considerable differences between humans and rodent species. Cyp7a1 is the critical regulatory enzyme in rodents that synthesizes primary bile acids. It interacts with the liver X receptor (LXRα), a relationship that does not occur in humans. The Cyp8B1 promoter in human subjects can bind with the FXR [69,70] (Table 1).

**Table 1:** Overview of some metabolites produced by microbiota.

Metabolites	Produced Metabolites	Gut Microbes	Functions	Ref.
Short-chain fatty acids (SCFA)	Acetate, Propionate, Butyrate.	*Bacteroidetes*, *Firmicutes*, *Campylobacter jejuni*, *Staphylococcus aureus*, *Bifidobacterium* sp. *Coprococcus*, *Clostridium*, *Roseburia*, *Faecalibacterium*	Cell signaling–mediated host metabolic pathway regulation. Immunomodulation. Maintenance of energy homeostasis. Increased glucose tolerance and insulin sensitivity. Osmotic balance regulation. Fat oxidation. Defense against pathogens. Intestinal permeability regulation.	[71]
Bile acid metabolites (BA)	Cholic acid, Deoxycholic acid, Chenodeoxycholic acid, Taurocholic acid, Lithocholic acid, Glycocholic acid.	*Bifidobacterium*, *Bacteroides*, *Clostridium*, *Lactobacillus*, *Enterobacter*	Intestinal barrier regulation. Activate host nuclear receptors and cell signaling pathways. Exhibit antimicrobial effects. Lipid absorption regulation.	[72]
Aromatic amino acids (AAA)	Indoleamine 2,3-dioxygenase (IDO1), Aryl hydrocarbon receptor (AhR) Tryptamine (TA), Indole-3-acetamide (IAM), Tryptophan 2-monooxygenase (TMO).	*Escherichia coli*	Alter the host’s immune responses. Managing immune responses and inflammation. Influencing cytokine production. Development of Treg cells.	[72]
Indole derivatives	Indole, Indole-3-propionic acid, 5-hydroxyl indole, indoxyl sulfate, N-acetyltryptophan, indoxyl sulfate, Serotonin, Melatonin, Melatonin 6-sulfate.	*Escherichia coli* *Clostridium sporogenes*	Antioxidant. Neuroprotection and cytoprotection. Intestinal barrier regulation. Regulation of endothelial dysfunction. Regulation of cardiovascular disease.	[71]

### 2.2. Microbial Metabolites and Cancer

Increasingly, the GM is seen as significant in health and disease. Cancer is associated with particular bacteria and viruses that can lead to cell dysplasia and tumorigenesis. *Salmonella typhi* and *Helicobacter* spp. are recognized as oncogenic bacteria in biliary cancer, with *Helicobacter pylori* implicated in gastric cancer. While chronic inflammation typically contributes to carcinogenesis, some bacteria, such as *H. pylori*, can directly harm genetic material and interfere with essential signaling mechanisms that manage the proliferation of mucosal cells [73].

#### 2.2.1. Impact of Microbial Metabolites on Cancer Therapy

In the colon, bacteria use processes beyond fermentation, including aerobic respiration with nitrates and sulfates as electron receptors. Microbial metabolites like TMAO, lactacystin, and SCFAs (butyrate, acetate, propionate) aid nutrition and support intestinal epithelial cell growth. Butyrate improves intestinal integrity and aids tissue repair. Microorganism-produced metabolites influence the immune system; low SCFA levels decrease regulatory T-cell counts and increase inflammation. These metabolites also affect cancers like colon, lung, and prostate by interacting with tumor cells. Inflammation activates immune responses, producing cytokines like TNF and interleukins, which can lead to diseases. The relationship between intestinal microbes and hosts is mutual; disruptions can cause inflammatory issues [74].

#### 2.2.2. Impact of Microbial Metabolites on Inflammation, Necrosis, and Apoptosis

Cancer, a major cause of death, has seen increased recognition recently. Innovative treatments like antibody-drug conjugates and immunotherapy have emerged. Still, their success is hindered by drug-resistant tumor cells and the complex TME. The TME’s disorganized vascular system can limit chemotherapy flow, while immunosuppressive cells reduce immune responses to immunotherapy [75]. Microbial metabolites can both promote and inhibit cancer; for example, gut bacteria converting primary to secondary bile acids disrupt NK T-cells, fostering tumors. *H. pylori*’s cytotoxin triggers DNA breaks linked to gastric cancer. Conversely, *Lactobacillus reuteri* produces reuterin, which targets proteins in colon cancer cells. Focusing on these metabolites could enhance treatment outcomes by increasing effectiveness and reducing side effects [17].

**Figure 2:**
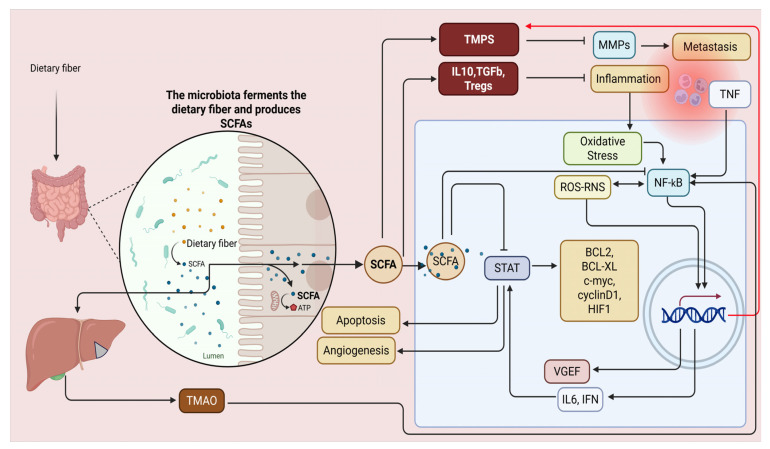
Examining SCFAs in combating NF-κB-associated inflammation and cancer risk is crucial due to the complexity of inflammatory pathways in carcinogenesis. NF-κB up-regulates matrix metalloproteinases (MMPs), aiding metastasis, enhances VEGF for angiogenesis, and affects STAT signaling for cell proliferation while inhibiting apoptosis—key cancer progression traits. SCFAs counteract these by promoting T-cell differentiation into regulatory cells, stimulating anti-inflammatory cytokines, and upregulating tissue inhibitors of metalloproteinases (TIMPs) to reduce metastasis and block NF-κB and STAT pathways. Activation of NF-κB and the NLRP3 inflammasome is essential but concerning for TMAO-induced vascular calcification. The NLRP3 inflammasome’s role in inflammation complicates understanding chronic inflammation’s impact on blood vessels. This figure was created with BioRender software version 04.

The potential of microbial metabolites lies in their ability to function as complementary therapeutic agents, thereby enhancing current medical methodologies. Butyric acid, produced by gut bacteria, modulates T-cell receptor pathways to enhance the release of cytokines with antitumor properties. The presence of butyric acid significantly strengthens immune cells’ capacity to combat tumors, improving anti-programmed PD-1 immunotherapy outcomes. Succinic acid generated by the metabolism of *F. nucleatum* interferes with IFN-β production. It restricts the infiltration of CD8+ T-cells into the TME. Consequently, the body’s defense against the tumor is weakened [76]. The effects of microbial metabolites on tumor therapy can be both advantageous and detrimental. Understanding the dual influence requires explaining its mechanism and analyzing the environment where it takes effect. SCFAs in differing quantities produce distinct results in cancer therapy. Tumor treatment results vary according to the levels of SCFAs administered. High SCFA concentrations in non-alcoholic fatty liver disease may lead to hepatocellular carcinoma if they go beyond the host’s tolerance threshold. SCFAs, in proper quantities, play a crucial role in preventing the growth of colorectal cancer. The relationship between microbial metabolites and tumors is not straightforward; their effects change with concentration [17,77] (Figure 2).

### 2.3. Microbiota Metabolites and Tumor Progression

During their advancement, tumor cells strategically influence essential signaling pathways in their structure and neighboring cells, thereby cultivating a microenvironment conducive to their growth and metastasis. Hepatocyte growth factor is secreted by SNU-484 cells, which also show high levels of its receptor Met. Autocrine activation boosts mitogen-activated protein kinase (MAPK) signaling, which contributes to the progression of gastric cancer. Colorectal cancer cells release extracellular vesicles rich in miR-181a-5p, which activate the IL-6/STAT3 signaling pathways in hepatic stellate cells. The interaction of colon cancer cells with hepatic stellate cells alters the TME and promotes liver metastasis [78,79]. Consequently, cancer progression leads to ongoing changes in the TME and signaling pathways. In the TME, metabolites produced by the microbiota may accumulate and bind to specific receptors, impacting protein function, signaling pathways, gene expression, and cytokine concentrations. The Wnt/β-catenin signaling pathway is activated when lactate from microbes binds to the GPR81 receptor on cervical squamous cells [80,81]. The expression of Fut8, which codes for α-1,6 fucosyltransferase, is elevated by Wnt pathway activation. Enhanced fucosylation in vaginal epithelial cells plays a crucial role in preventing cervical cancer from progressing. Cancer advancement is primarily driven by microbial metabolites that modify the control signaling pathways and TME [82].

#### Various Signaling Pathways, Tumor Progression, and Metabolite Microbiota

Tumor progression is linked to signaling mechanisms controlling cell growth and apoptosis in immune and tumor cells. Microbiota metabolites affect cancer advancement by activating or inhibiting these pathways. The MAPK signaling pathway is crucial for tumor progression, cell death, metastasis, and maintaining cancer stem cells. It involves proteins like rapidly accelerated fibrosarcoma (RAF), MEK, and extracellular signal-regulated kinase (ERK) in mammals [83]. Activated MAPKs phosphorylate proteins that affect gene expression related to cancer. Four main MAPK pathways involve p38 MAPK, c-Jun N-terminal kinase, ERK5, and ERK1/2. Microbial metabolites regulate MAPK pathways: Kynurenic acid reduces colon cancer growth by lowering ERK1/2 phosphorylation. At the same time, LCA activates it to promote IL-8 secretion and speed up cancer development. Manumycin A from *Streptomyces* sp., a soil bacterium also found in the human GM, shows the historical connection between humans and soil microbes [72].

Manumycin A presents a compelling strategy in the fight against cancer by effectively inhibiting Ras farnesylation. This critical process allows Ras proteins to anchor to the cell membrane. This inhibition is crucial as it disrupts the activation of the Ras/Raf/ERK1/2 signaling pathway, which plays a significant role in driving cancer progression. In castration-resistant prostate cancer cells, where traditional therapies often fail, Manumycin A impedes this vital signaling route and curtails the formation and release of exosomes—tiny vesicles that can facilitate tumor growth and metastasis [84]. Manumycin A offers a dual approach to combat aggressive cancer forms by blocking these pathways and mechanisms. With promising evidence supporting its efficacy, incorporating Manumycin A into treatment regimens could transform outcomes for patients facing difficult-to-treat cancers. Thus, this compound stands out as a critical player in advancing targeted therapies aimed at crippling cancer’s ability to thrive and spread. Exosomes appear to aid tumor growth and metastasis. SCFAs bind to FFAR3, inhibiting MAPK signaling in breast cancer cells and promoting a non-invasive phenotype [84,85].

Urolithin A (UA) suppresses MAPK activation in LPS-stimulated macrophages by blocking p38 and JNK phosphorylation, reducing pro-inflammatory factors, and slowing tumor growth. In colon cancer cells, butyrate triggers MAPK signaling, decreasing proliferation and increasing apoptosis by enhancing TLR4 expression. Stimulating the phosphatidylinositol-3-kinase (PI3K) and the serine/threonine protein kinase (Akt) pathway enhances cell proliferation and survival; inhibiting it induces apoptosis [72]. PI3K produces phosphatidylinositol (3, 4, 5)-trisphosphate when detecting survival signals. PDK1 and mTOR activate Akt, altering proteins linked to apoptosis. SCFAs in colon cancer cells suppress PI3K/Akt signaling to induce apoptosis by reducing BAD phosphorylation, promoting cytochrome c release from mitochondria, and increasing caspase-3 levels. KYNA reduces Akt phosphorylation to prevent colon cancer cell proliferation; UA acts similarly on the PI3K/Akt/mTOR pathway in pancreatic ductal adenocarcinoma (PDAC), combating cancer by inhibiting Akt phosphorylation and p70 ribosomal S6 protein kinase (p70S6K). UA also reduces inflammation in chronic alcoholic pancreatitis. LCA upregulates miR21 in colon cancer cells, inhibiting phosphatase PTEN. S-equol hinders breast cancer MCF-7 cell growth by enhancing miR-10a-5p expression that targets PIK3CA’s 3′ UTR to repress the PI3K/Akt pathway [86,87].

**Figure 3:**
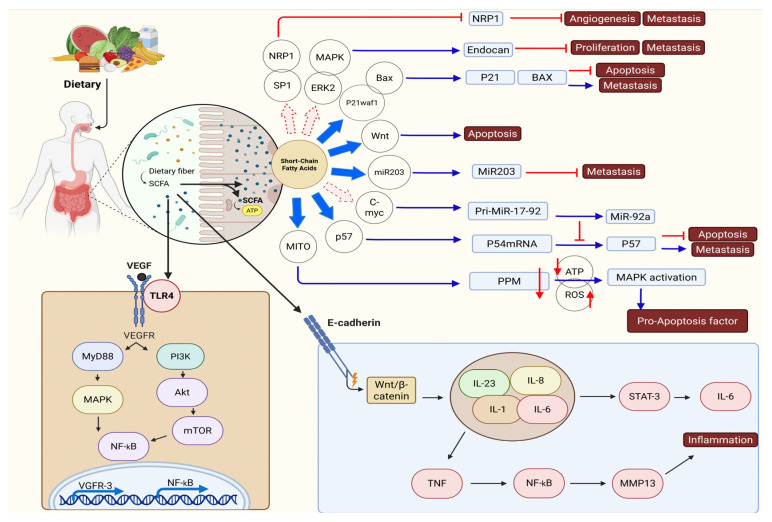
The effects of short-chain fatty acids (SCFAs) like acetate, propionate, and butyrate on the tumor immune microenvironment (TIME). Erk activation enhances the levels of vimentin and N-cadherin, thereby driving the occurrence of EMT. The presence of microorganism-associated molecular patterns (MAMPs) on microbes allows macrophages to identify them using Toll-like receptors (TLRs). The production of reactive oxygen species (ROS) can occur in macrophages, or the activation of different signaling pathways can lead to the release of proinflammatory cytokines, including IL-1, IL-6, IL-8, IL-23, and TNF. The activation of STAT3 and NF-κB signaling pathways by proinflammatory cytokines triggers the expression of the c-myc oncogene and MMP13, which subsequently contributes to epithelial-mesenchymal transition (EMT), persistent inflammation, and ultimately the development of cancer. At the same time, FadA and BFT, acting as virulence factors, can hinder E-cadherin functionality, which activates the β-catenin/Wnt signaling pathways and subsequently leads to the activation of the STAT3 and NF-κB pathways. Short-chain fatty acids (SCFAs) promote the production of IL-22 in CD4+ T-cells and innate lymphoid cells (ILCs) through mechanisms involving HIF-1α and AhR, as well as the participation of Stat3 and mTOR. This figure was generated using BioRender software version 04.

Multiple signaling routes are linked to cancer advancement, notably the JAK/STAT3 pathway, which is crucial for inflammation and promotes tumor growth. *Faecalibacterium* metabolites hinder the development of breast cancer cells through the suppression of IL-6/STAT3 signaling. Evidence suggests that LCA contributes to cancer by lowering the performance of STAT3. The effects of STAT3 signaling on cancer are dual, encompassing both promotion and suppression. The interaction between Rac1, a Ras-related C3 botulinum toxin substrate, and p21-activated kinase 1 (PAK1) governs the actin polymerization process, which is integral to cell division and mobility [72]. The signaling pathway involving Rac1 and PAK1 frequently exhibits overactivation across various cancer types. The action of UA leads to a reduction in Rac1 activity and PAK1 phosphorylation, which contributes to the inhibition of cancer. By regulating the Nrf2 signaling pathway, ellagic acid and its metabolite UA could play a role in preventing colon and liver cancer by reducing oxidative stress and inflammatory damage. The Hippo/YAP pathway promotes cancer growth. When SCFAs bind to FFAR2 on breast cancer cells, they inhibit Hippo/YAP signaling, elevate E-cadherin levels, and stop the cells from acquiring invasive properties [72,88] (Figure 3).

## 3. Intestinal Roundworms Promote Microbial Metabolites in Tumor Progression

Cancer has the highest mortality and morbidity rates among diseases that threaten human health. Despite scientific advancements and extensive research, it remains a significant health concern. This has led scientists to increasingly explore new solutions to this critical issue [89]. Parasitic nematodes have long been harmful to humans and some animals. In many regions of the world, these parasitic diseases remain prevalent, with statistics indicating that approximately 24% of gastrointestinal infections are caused by nematodes [89,90].

Millions of people are affected by roundworms every year, and these devastating effects can be seen in both personal health and economic aspects [91]. Inhibition of immune responses and subsequent modulation of these responses are among the extraordinary abilities of nematodes [92]. The findings suggest that certain nematodes and their metabolites may positively influence the healing process of autoimmune diseases and allergies by affecting the host immune system [93,94]. According to certain studies, increased IL-10 and TGF-β from Tregs inhibit Th1 and Th2 cell activity, helping the parasite evade expulsion and protecting the host from inflammation-related damage [95].

Since most approaches to autoimmune diseases focus on managing symptoms, it is inaccurate to refer to these as “treatments.” The use of helminth therapy to control autoimmune diseases such as ulcerative colitis, Crohn’s disease, or multiple sclerosis is one of the methods that has recently attracted much attention. Several studies have examined the association between the use of nematodes such as *Trichuris suis* and *Necator americanus*, which help to improve the course of autoimmune diseases [96,97,98,99]. A correlation exists whereby individuals with autoimmune diseases are more susceptible to cancer than individuals who are in good health. The prevalence of intestinal carcinoma and epithelial cell dysplasia is significantly elevated in patients suffering from inflammatory bowel disease when compared to individuals without this condition [100].

### 3.1. Intestinal Roundworms in Tumor Progression

In a murine model, the presence of an inflammatory milieu during colitis influences nematodes’ survival rates, immunomodulatory characteristics, and immunogenic potential. Colitis creates an inflammatory setting that significantly impacts nematodes’ viability, capacity to influence immune functions, and immunogenic traits, particularly observed in studies using mice. This analysis brings to light the nuanced relationship between gastrointestinal inflammation and the conduct of these parasitic organisms in a living host [101,102]. Some parasitic worms, notably *Schistosoma haematobium* and *Opisthorchis viverrini*, are associated with tumor promotion. [103,104]. Products from small intestinal nematodes can lead to the over-proliferation of normal intestinal epithelial cells. Increasing immune system responses and disrupting the progression of some cancers can be seen following infection with some nematodes, such as *Nippostrongylus brasiliensis* [105].

The pro-apoptotic activity linked to the mitochondrial pathway has been demonstrated in the excretory-secretory (ES) products of *Trichinella spiralis* muscle larvae. In a study involving small-cell lung cancer cells, co-culturing with the ES resulted in upregulation of several pro-apoptotic genes, including Bax, Cyt-C, Apaf-1, caspase-9, and caspase-3. In contrast, there was a noted downregulation of anti-apoptotic genes, specifically Bcl-2 and Livin. This suggests that the ES from *T. spiralis* ML may significantly promote apoptosis in cancer cells by modulating the expression of key apoptotic regulators [106].

In Table 2, different nematodes produce suppressor molecules with various functions. Some of these molecules can reduce the intensity of the Th2 response. Suppression of protective mechanisms is another function of these molecules. Another characteristic of these molecules is their ability to inhibit protective mechanisms. In a study, the effect of nematode infection on EL4 metastasis was examined in three groups of mice: one group co-treated with the nematode, another group pre-treated with the nematode, and a third group untreated. The results indicated that infected mice had increased serum concentrations of TGF β [107].

Research evaluating the anticancer impact of *Toxocara canis* excretory-secretory Troponin protein peptide (ES TPP) in vitro demonstrated that a concentration of 100 μg/mL significantly modified gene expression in cancer cells of the gastrointestinal tract and liver [107]. The suppression of pro-inflammatory cytokines by parasites leads to the induction of anti-inflammatory cytokines [108,109]. Some helminths can directly interact with immune cells, such as eosinophils, macrophages, and natural killer (NK) cells. This interaction activates these cells and directs them to the site of inflammation. Additionally, immune cells can exhibit antitumor activity following this interaction [110,111,112,113]. Helminths can reduce chronic inflammation and prevent tumor development by inducing changes in the immune system [114,115]. Angiogenesis in cancer cells is crucial for their survival and growth. Helminths inhibit the angiogenesis factor, thereby preventing the development of cancer cells [116,117].

#### 3.1.1. *Echinococcus* *granulosus*

*Echinococcus granulosus* is a tapeworm that belongs to the Taeniidae family. This cestode primarily exists as an adult in carnivorous animals. Herbivores, such as sheep, serve as intermediate hosts and play a crucial role in completing the transmission cycle. The eggs of this cestode are found in the feces of carnivorous hosts. When herbivorous hosts ingest these eggs, they develop into cysts. Carnivores become infected with this parasite by consuming meat contaminated with these cysts [118,119,120]. Various antigens have been isolated from *E. granulosus,* and their anticancer potential has been evaluated, including Antigen B (AgB), Antigen 1 (Ag1), and Antigen 5 (Ag5). Evidence suggests that multi-subunit antigen AgB can trigger humoral and cellular immune responses that could benefit anticancer activity [118,119,120,121,122,123].

#### 3.1.2. *Trichinella spiralis*

*Trichinella spiralis* infects many mammals, including carnivores, omnivores, and humans. In the life cycle of this parasite, the adult worm produces larvae through sexual reproduction in the host’s small intestine. The larvae penetrate the intestinal tissue, enter the bloodstream, and travel to various tissues, becoming cysts. Eating muscle infected with cysts can lead to infection of a new host [124,125,126]. Inducing immunological reactions, activating the apoptosis pathway in cancer cells, and consequently preventing the growth of cancerous tumors are among the events that occur following the entry of antigens derived from *T. spiralis* into the body [125,127,128]. TSL-ES excretory-secretory products of *T. spiralis* are antigens that modulate TME and exhibit antitumor activity [125]. Additionally, the activation of dendritic cells and triggering an immune response against cancer cells results from *T. spiralis* antigens entering the body [129,130].

#### 3.1.3. *Toxocara canis*

*Toxocara canis* is a zoonotic parasite that can infect dogs and humans. Humans can contract toxocariasis by ingesting the parasite’s eggs, typically through contact with contaminated soil or materials [131,132]. Toxocariasis in humans mainly affects organs such as the eyes, liver, and lungs [133]. Evidence suggests that toxocariasis produces antigens that can modulate the immune response, potentially reducing the risk of certain cancers, including colon and breast cancer. This occurs through the increased activity of macrophages, DCs, NK cells, and T2 immune responses, which ultimately inhibit the progression of tumor cells (Table 2) [134]. In a study investigating the effects of *T. gondii* and *T. canis* egg antigens on transplanted WEHI-164 fibrosarcoma in a mouse model using BALB/c mice, it was found that these antigens possess inhibitory properties on tumor cells [135,136].

#### 3.1.4. *Taenia solium*

Research findings indicate that recombinant *T. solium* calreticulin (rTsCRT) has potent antitumor properties. In studies involving the SKOV3 and MCF7 cell lines, rTsCRT demonstrated a dose-dependent reduction in cell viability and colony-forming ability, particularly affecting cancer stem-like cells. The antitumor effects were linked to its interactions with scavenger receptors; blocking these receptors reversed the reduction in cell viability. Therefore, rTsCRT holds promise as a potential therapy for breast and ovarian cancers (Table 2) [137,138,139,140].

Helminths have special abilities to maintain their survival in the host body. One of these extraordinary abilities is to evade the host’s immune system [141,142,143]. Numerous studies have explored the effectiveness of helminths in treating various diseases, including multiple sclerosis, inflammatory bowel disease, celiac disease, atherosclerosis, and non-alcoholic fatty liver disease, as well as several types of cancer, such as colorectal cancer [106,144,145,146,147,148,149,150,151]. Several helminths can affect tumor growth, including *Clonorchis sinensis, Opisthorchis viverrini*, and blood flukes such as *Schistosoma haematobium, Schistosoma japonicum*, and *Schistosoma mansoni* [152,153,154]. A study found that *Eimeria granulosa* can suppress tumors by inducing a Th1 immune response [155,156]. *Echinococcus multilocularis* larval (E/S) antigens can induce the transformation of CD4 (+) T-cells into Foxp3 (+) Tregs in vitro through a TGF-beta-dependent mechanism [157]. Extracellular vehicles (EVs) play a crucial role in parasites’ interaction with cancer cells. A study revealed that *Nippostrongylus brasiliensis* protects against intestinal inflammation in mice by secreting EVs [158] (Table 2).

**Table 2:** Antitumor activity of different intestinal roundworms.

Parasite	Cancer	Mechanism of Action	Reference
*Echinococcus granulosus*	Breast and colon cancer	Production of antibodies for the recognition of tumor cells	[118,119,120,121,122,123]
	Fibrosarcoma	Not clear	
*Taenia crassiceps*	Colitis-associated colorectal cancer	Decrease recruitment of inflammatory monocytes and inflammation in the colon.	[159]
*Taenia solium*	Breast and ovarian cancers	Recombinant *T. solium* calreticulin (rTsCRT) has potent antitumor therapy	[137,138,139,140]
*Toxocara canis*	Colon and breast cancer	Toxocariasis produces antigens that can modulate the immune response, potentially reducing the risk of certain cancers.	[135,136]
		Increased activity of macrophages, dendritic cells (DCs), natural killer (NK) cells, and T2 immune responses.	
*Toxoplasma gondii*	Melanoma	Activation of CD8^+^ and NK cells and expression of MHC-I and MHC-II in APC	[159]
	Fibrosarcoma	Increase in the activity of cytotoxic T-cells	[159]
	Melanoma and lung cancer	Suppression of neovascularization via induction of hypoxia and avascular necrosis	[159]
*Trichinella spiralis*	Melanoma	Reduction of lung metastasis through CXCL9, CXCL10, IL-4, CXCL1 and CXCL13	[124,125,126]
	Human hepatoma cell line (HT402) and human chronic myeloid leukemia cell line (K562)	Arrested in the cell cycle in G1 or S phase	[124,125,126]
*Trypanosoma cruzi*	Breast and colon cancer	Activation of CD4^+^ and CD8^+^ cells and production of antibodies against cancer cells	[159]
	Experimental breast adenocarcinoma	*Trypanosoma cruzi* calreticulin as a revealer of the presence of tumor cells in the immune system	[159]
	Mammary cancer	Inhibition of proliferation and migration of endothelial cells	[159]
	Melanoma	J18 recombinant protein induces apoptosis through caspase 3	[159]

### 3.2. Intestinal Roundworms and Bacterial Metabolism

With significant effects on host health, parasitic roundworms (intestinal nematodes) and the gut microbial population have a dynamic and complicated connection within the complex intestinal environment of humans. Numerous processes underlie this connection, including the direct impacts of worms on bacterial metabolism as well as the metabolic alterations that bacteria experience in response to worm presence [160]. By secreting various bioactive compounds, intestinal roundworms like *Heligmosomoides polygyrus* and *Necator americanus* drastically change the gut environment. To induce Th2 and Treg immunological responses, the worms first modulate the immune system. This immune activation produces specific cytokines, including IL-4, IL-5, IL-10, and IL-13, each influencing bacterial metabolism in distinct ways. For example, IL-13 causes goblet cells to produce more mucin, which gives bacteria that break down mucin, such as *Akkermansia muciniphila*, a rich food supply [161].

Additionally, the worms release specific enzymes that alter the structures of dietary macromolecules, such as glycosidases and metalloproteinases. These enzymes increase the accessibility of nutrients for gut microorganisms by breaking peptide and glycosidic bonds. In particular, MUC2 mucins are broken down by worm-derived enzymes, producing oligosaccharides that encourage the development of specific bacterial species [162]. Changes in the gut environment’s physicochemical composition are also quite significant. The worms change the pH values in their surroundings by secreting substances like organic acids and ammonia. According to studies, infection with *Nippostrongylus brasiliensis* can lower the luminal pH by up to 1.5 units, which favors acidophilic bacteria (such as *lactobacilli*) while suppressing species that are sensitive to acid [162,163].

The GM uses complex metabolic strategies to adapt to these changes. Increased synthesis of SCFAs such as butyrate, propionate, and acetate is a noteworthy adaptation, mainly due to the accelerated development of the *Lachnospiraceae* and *Ruminococcaceae* families. Butyrate works as a crucial signaling chemical through various pathways: blocking histone deacetylases (HDACs), activating G protein-coupled receptors (GPCRs), and acting as an energy source for colonic epithelial cells [164]. The metabolism of amino acids undergoes another meaningful metabolic change. When worms are present, bacteria like *Bacteroides vulgatus* alter tryptophan metabolism to create indole derivatives. These substances, especially indole-3-acetate, activate the aryl hydrocarbon receptor (AhR) and significantly impact host immunity and metabolism [165].

This interaction’s modulation of bile acid metabolism is one of its most intriguing features. The worms’ inhibition of bacterial 7α-dehydroxylase activity changes the primary-to-secondary bile acid ratio. This alteration inhibits the carcinogenic deoxycholic acid formation while raising anti-inflammatory ursodeoxycholic acid levels [166]. There are significant health effects from these metabolic changes. The interactions can lower calprotectin levels, an indicator of inflammation, by as much as 70% in inflammatory bowel illnesses. By blocking Wnt/β-catenin signaling and triggering apoptosis, the microbial metabolites may stop the development of gastrointestinal malignancies. In metabolic diseases, the metabolic alterations also enhance insulin sensitivity [161].

Targeted helminth therapy, metabolite-based therapies employing substances like sodium butyrate, and sophisticated microbiome interventions are some therapeutic uses for these discoveries. Nonetheless, there are still issues with standardizing treatment protocols and interindividual response heterogeneity [167,168]. This study area has much potential, especially in light of recent developments in omics technology. Significant advances in comprehending and using these intricate relationships are anticipated from future multidisciplinary collaboration, which might transform how we treat several illnesses over the next ten years. Translating these discoveries into therapeutic applications will need the confluence of immunology, microbial ecology, and systems biology [166,169,170].

### 3.3. Change of Microbial Metabolites by Intestinal Roundworms

Through a complicated web of interactions with the host microbiota, intestinal roundworms cause significant alterations in the gut microbial metabolite composition. These changes are brought about by some interrelated processes that provide the intestinal lumen with its distinct metabolic environment [169]. Helminths release immunomodulatory substances like ES-62 and HpARI at the molecular level, which tilt host immunity in favor of a Th2 response. This change increases the production of specific cytokines, such as IL-4 and IL-13, which modify the expression of genes in bacteria involved in the metabolism of carbohydrates. Interestingly, these cytokines increase the activity of glycosidase in bacteria such as *Bacteroides thetaiotaomicron*, which results in a more effective breakdown of complex polysaccharides and a higher production of SCFAs [171,172].

The most noticeable change is the sharp rise in butyrate production, mainly from the *Ruminococcaceae* and *Lachnospiraceae* groups. Several causes contribute to this fivefold elevation: Worm-mediated pH lowering produces ideal circumstances for these bacteria, helminth excretory products directly influence bacterial fermentation genes such as butyryl-CoA: acetate CoA-transferase, and worm-induced mucus secretion supplies an abundance of complex carbohydrates [164,173]. By secreting specialized proteases (like M13 metalloproteases) that degrade dietary proteins into smaller peptides and free amino acids and by modifying host immune responses that change amino acid availability, helminths also significantly impact amino acid metabolism. These modifications eventually increase the synthesis of indole derivatives, such as indole-3-acetate and indole-3-aldehyde, which are crucial for immunological control [174].

The metabolism of bile acids is also significantly altered. Helminths alter the primary-to-secondary bile acid ratio by blocking bacterial 7α-hydroxylase activity, which lowers pro-inflammatory DCA and raises anti-inflammatory UDCA. By activating nuclear receptors FXR and TGR5, these changes impact not just the digestion of fat but also systemic metabolism [175,176]. It is interesting to note that these metabolic alterations change with time. Increased generation of lactate and succinate during the acute infection phase (1–2 weeks) indicates a change in bacterial metabolism toward anaerobic processes. The profile shifts toward higher SCFAs and lower inflammatory metabolites by the chronic phase (>4 weeks), indicating complex host, worm, and microbiota adaptability [166,177].

There are several physiological repercussions. Locally, tight junction proteins such as occludin and claudin are upregulated by elevated butyrate, strengthening the gut barrier. Systemically, these modifications enhance insulin sensitivity, lower inflammation, and modify immunological responses. Notably, specific metabolic alterations endure even after the removal of worms, indicating the formation of “metabolic memory” within the GMs [178]. There are significant therapeutic applications for comprehending these processes. To treat inflammatory bowel illnesses, metabolic syndrome, and even neurological problems, current research investigates indole derivatives as dietary supplements or the combination of certain probiotics with helminth-derived components. Nonetheless, issues with interindividual variability and uniformity of treatment protocols still need to be looked at further [168,179].

### 3.4. Relationship Between GM and Roundworms in Inflammation, Necrosis, and Apoptosis

The complex immunomodulatory network produced by the dynamic interaction of intestinal roundworms (nematodes) and the GM significantly impacts inflammatory responses. Roundworm infections, especially those caused by *Ascaris lumbricoides* and *Enterobius vermicularis*, cause significant alterations in gut microbes’ ecology through several interrelated processes. By directly changing the gut microenvironment, these parasites’ excretory/secretory (ES) products promote anti-inflammatory bacterial taxa while inhibiting pro-inflammatory ones. One crucial mediator is the Th2 immune response from roundworms, which is marked by increased production of IL-4 and IL-13. This response increases mucus production by goblet cells and changes the physicochemical environment of the gut lumen [180,181].

Mucus-using bacteria like *Akkermansia muciniphila* thrive in this altered environment, while roundworm ES products inhibit inflammation-linked Enterobacteriaceae growth. Host antimicrobial peptides are released when parasites interact with the intestinal lining, affecting the makeup of microorganisms. Roundworms create an immunosuppressive environment that promotes *Lactobacillus* and *Bifidobacterium* growth by inducing Treg cell proliferation via TGF-β and IL-10 release; these commensals enhance anti-inflammatory activity through SCFAs, notably butyrate’s HDAC inhibition [182]. Parasite-specific variations exist: *Trichuris trichiura* infections usually reduce pathobionts and boost diversity, whereas high *Ascaris* loads may cause *Bacteroides* overgrowth. Roundworm-microbiota interactions significantly influence inflammatory pathways via mechanisms like (1) roundworm glycans binding host receptors to alter DC function, (2) microbial SCFAs promoting Treg differentiation through GPR43/109a signaling, and (3) parasite-induced IL-22 enhancing epithelial barriers to prevent bacterial translocation. This interaction creates equilibrium during chronic infections; parasite chemicals like ES-62 modify dendritic cells for hyporesponsiveness toward antigens [20]. Despite allowing parasite persistence, low-grade inflammation surprisingly protects against inflammatory illnesses by activating macrophages through the microbiome. Species-specific characterization remains needed due to regulatory heterogeneity among nematodes. Still, this balance highlights the therapeutic potential of leveraging roundworm-microbiome interactions for managing inflammatory diseases [20,183].

**Figure 4:**
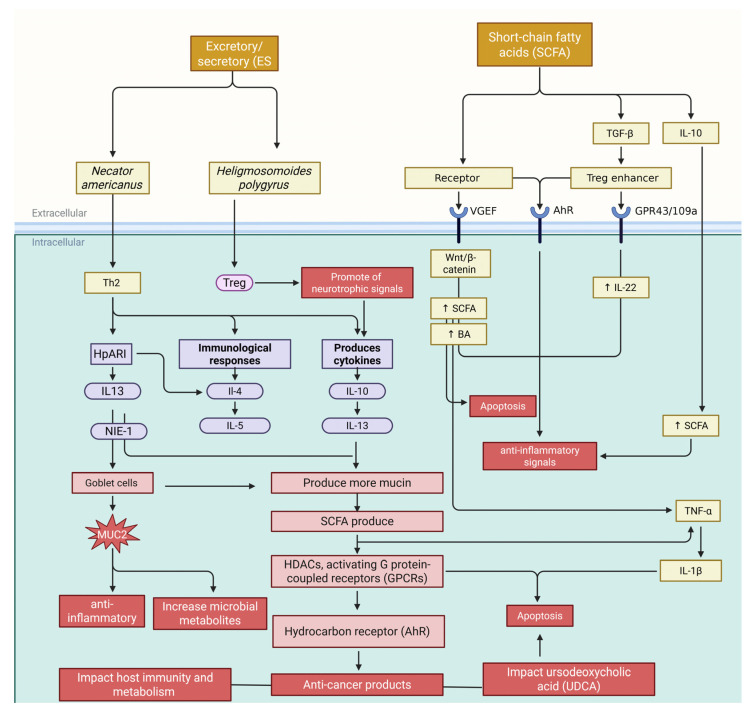
The intricate relationship between the GM and roundworms presents a dual-edged sword in the context of inflammation, necrosis, and apoptosis. While excretory/secretory (ES) products from roundworms encourage anti-inflammatory bacterial taxa and suppress pro-inflammatory ones, the complexity deepens with the Th2 immune response. This response, characterized by elevated levels of IL-4 and IL-13, stimulates goblet cells to increase mucus production, altering the gut lumen’s environment. Such changes support the proliferation of regulatory T-cells (Tregs) via TGF-β and IL-10 release, promoting an anti-inflammatory milieu strengthened by short-chain fatty acids like butyrate. However, this balance is precarious; any breakdown of the intestinal barrier can allow harmful microbial compounds such as lipopolysaccharides to infiltrate systemic circulation. The ensuing cascade involves reactive oxygen species and pro-inflammatory cytokines like TNF-α and IL-1β being released through TLR4 signaling in macrophages. Despite this turmoil, roundworms contribute to regulating immune cell apoptosis at a cellular level; they help sustain Treg survival through IL-10 and TGF-β signaling during infection. Ultimately, while specific mechanisms foster immunological tolerance and curb inflammation, they also highlight vulnerabilities that could exacerbate systemic inflammatory responses if not carefully modulated. This figure was generated using BioRender software version 04.

The GM and roundworms significantly influence tissue necrosis in hosts (Figure 4). Roundworm infections, like those from *Nippostrongylus brasiliensis* or *Strongyloides stercoralis*, can indirectly cause necrosis by altering gut microbes and directly through parasitic activity [184]. Parasite-secreted proteases break down the extracellular matrix, leading to localized injury while disrupting microbial balance by reducing beneficial bacteria such as *Lactobacillus* and *Bifidobacterium*. This shift often leads to an overgrowth of harmful bacteria like *C. difficile* and *E. coli*, which release toxins that damage epithelial cells and trigger immune overreactions, worsening necrosis. Toxins from *C. difficile* can damage the cytoskeleton, causing cell death. The Th2 immune response induced by roundworms may unintentionally promote necrosis by suppressing antimicrobial peptides via elevated IL-4 and IL-13 levels—this weakens the intestinal barrier, facilitating bacterial migration. When microbial compounds like LPS enter circulation due to barrier breakdown, they activate macrophages through TLR4 signaling, releasing ROS and pro-inflammatory cytokines that worsen oxidative stress and tissue damage. Chronic infections create a cycle: dysbiosis and inflammation lead to increased production of cellular debris. At the same time, a weakened barrier allows further bacterial invasion [184,185].

Some roundworms, like *Heligmosomoides polygyrus*, alter the microbiota to prevent necrosis by promoting *Akkermansia muciniphila*, which enhances mucus and barrier repair. Compounds like ES-62 from roundworms reduce tissue damage by inhibiting TLR-mediated inflammation. The balance of beneficial and harmful effects depends on the parasite species, infection severity, and host-microbiome baseline. The GM-roundworm axis in necrosis has a dual role: interactions may reduce necrosis via immunomodulation and barrier reinforcement or worsen tissue damage through dysbiosis and toxins [186,187]. Understanding these processes could lead to anti-parasitic or microbiome restoration therapies for necrosis-related diseases. This interaction balances tissue homeostasis and pathology in modifying apoptotic pathways in intestinal epithelium and immune cells during infections like those caused by *Heligmosomoides polygyrus* or *Nippostrongylus brasiliensis*. Parasites secrete excretory/secretory (ES) products that interact with immune cells to control apoptosis signals. At the same time, changes in gut microbes further refine these pathways, either stimulating or inhibiting apoptosis as needed [188,189].

In Figure 4, the roundworm-driven Th2 immune response increases IL-4, IL-13, and IL-10 levels. These cytokines downregulate Fas/FasL and TNF-α pathways, reducing pro-apoptotic signals in epithelial cells to enhance tissue healing and cell survival. For instance, IL-13 boosts STAT6 activity to suppress pro-apoptotic Bax and caspase-3 while elevating anti-apoptotic proteins like Bcl-2. This mechanism can shield damaged or precancerous cells from apoptosis but is crucial for maintaining epithelial integrity during infection. The GM influences these effects through microbial metabolites. The SCFAs like butyrate encourage apoptosis in injured epithelial cells by inhibiting HDAC and activating apoptotic pathways [190,191]. Conversely, LPS-mediated TLR4 activation triggers NF-κB-dependent survival signals that inhibit apoptosis in roundworm-induced dysbiosis. Roundworms modify the microbiome to favor bacteria such as *Akkermansia muciniphila*, enhancing mucosal barrier function. Roundworms and microbiota jointly regulate immune cell apoptosis; regulatory T-cells resist apoptosis due to IL-10 and TGF-β signaling during infections, fostering immunological tolerance. Some bacteria create an anti-inflammatory environment; for example, *Lactobacillus rhamnosus* upregulates FasL to induce Th17 cell apoptosis. However, persistent roundworm antigens may cause excessive lymphocyte apoptosis through PD-1/PD-L1 in chronic infections, impairing immunity [25,192].

Understanding these relationships has potential therapeutic applications. For example, by restoring butyrate levels, the probiotic *F. prausnitzii* may counteract roundworm-induced apoptosis suppression, and targeted blocking of parasite-derived anti-apoptotic molecules may improve the removal of cancer cells. However, because apoptosis plays two roles in immune control and tissue regeneration, it must be carefully managed to prevent unforeseen outcomes like worsened inflammation or poor parasite clearance. In conclusion, apoptosis is regulated by the GM-roundworm axis through a complex interaction between host cytokines, microbial metabolites, and parasite factors. In addition to influencing infection outcomes, this dynamic balance between cell survival and death also affects more general pathologies like cancer and autoimmune illnesses, suggesting that it may be a promising target for therapy [193] (Figure 4).

### 3.5. Intestinal Roundworms—Gut Microbial Metabolites for Prevention and Treatment of Cancer

A new cancer immunotherapy paradigm involves metabolic and immunological signals from the interaction between the GM, parasitic roundworms (helminths), and their human host. Helminth-secreted substances and their effects on gut microbes drive these interactions, leading to novel cancer treatments. Chemicals from worms like *Heligmosomoides polygyrus* shift the immune response to an anti-inflammatory profile via cytokines IL-4, IL-5, IL-10, and IL-13. These cytokines enhance regulatory T-cells (Tregs) by blocking NF-κB and MAPK pathways, reducing pro-inflammatory cytokines like TNF-α. IL-13 increases mucin production in goblet cells, strengthening the gut’s protective layer [193]. This creates a favorable environment for bacteria from the *Lachnospiraceae* and *Ruminococcaceae* groups that produce cancer-fighting SCFAs like butyrate. Butyrate acts as a histone deacetylase inhibitor, enhancing tumor suppressor genes such as p21 and p53 by altering histone acetylation patterns while activating GPR109a receptors on immune cells for anticancer effects (Figure 4) [85].

Roundworms increase indole-3-carbinol (I3C) and IAA by fostering bacteria that produce indole and SCFAs. These substances have anticancer properties by attaching to aryl hydrocarbon receptors (AhR), which suppress pro-inflammatory responses, promote Treg differentiation, and boost detoxification enzymes [194]. Roundworms also promote mucin-degrading bacteria like *Akkermansia muciniphila*, preserving the intestinal barrier with metabolites like acetate and propionate. This barrier prevents endotoxins from causing oxidative stress and inflammation linked to cancer. Helminth-secreted substances like ES-62 prevent NF-κB activation, reducing pathogen-induced inflammation [195]. Bacteria such as *F. prausnitzii* produce polyamines that inhibit oxidative DNA damage and epigenetic alterations, while inducing autophagy to eradicate precancerous cells. These findings suggest new cancer therapies combining traditional treatments with probiotics containing SCFA-producing bacteria. For example, giving *Bifidobacterium longum* and *F. prausnitzii* probiotics alongside chemotherapy may improve effectiveness and reduce gastrointestinal side effects by fortifying the intestinal barrier against bacterial translocation and systemic inflammation while producing anticancer chemicals [196].

Using pure metabolites like sodium butyrate or indole-3-carbinol as supplements shows promise, especially rectal butyrate in halting colorectal cancer advancement in ulcerative colitis patients. These substances activate apoptosis and inhibit HDAC to kill precancerous cells. Combining immune checkpoint inhibitors with helminth-derived factors, such as ES-62, with anti-PD-1 medication improves antitumor responses and reduces chemotherapy-induced inflammation in animal melanoma models. Another approach is helminth-modified FMT, which uses healthy donor microbiota exposed to helminths for treating resistant gastrointestinal cancers. However, clinical translation faces challenges like individual GM differences affecting treatment efficacy and determining optimal dosage and treatment length to avoid infections or severe immunosuppression [197,198].

It is also essential to carefully assess any potential negative consequences. Even while studying helminths, which are usually not harmful, some people may nevertheless have gastrointestinal distress or allergic responses. Close observation is also necessary for the long-term impacts of microbiome changes.

## 4. Conclusions

This review focuses on clarifying how metabolites produced by GM influence cancer, particularly in terms of its progression and therapeutic approaches. The progression of cancer is affected by microbial metabolites, which change the TME and modify immune cell functions and cytokine concentrations. Various signaling pathways, including MAPK, PI3K/Akt, NFκB, and Wnt, are impacted by microbial metabolites, affecting cancer cells’ growth, apoptosis, and metastasis. The roles of secondary BAs, SCFAs, amino acid metabolites, and bacteriocins like LPS, nisin, and pyocyanin in the carcinogenesis and advancement of BC were assessed. The role of bacterial metabolites in affecting the success of BC treatments, including chemotherapy, targeted therapy, and immunotherapy, was also examined. Analyzing the microbiota before treatment helps oncologists understand tumor behavior and chemotherapy response, allowing for tailored treatment plans.

Exploring microbial metabolites’ role in cancer progression reveals their impact through pathways like TME modulation, signaling alterations, DNA repair enhancement, epigenetic modifications, and ROS production. Despite unidentified processes, understanding microbial metabolism of pharmaceuticals is crucial to tackle drug resistance and optimize cancer therapies. Microbial metabolites enhance treatments like radiotherapy, chemotherapy, and immunotherapy. Diet, probiotics, and prebiotics influence breast cancer treatment by fostering beneficial gut bacteria that convert fibers into phytoestrogens and SCFA with tumor-suppressing effects. Studying the synergy between gut metabolites and therapies could improve clinical safety and efficacy.

Alterations in host–microbiota interactions may increase cancer risk, as pathogens can reshape the microbiota. Future studies should clarify these causal relationships in carcinogenesis and identify underlying mechanisms. Microbes inhabit various body parts beyond the gut, affecting cancer progression and management significantly. Advances in metagenomics and systems biology enhance understanding of these relationships. Promising research includes next-generation probiotics for antitumor immunity and synthetic helminth analogs combined with immunotherapy or cellular therapy to tackle therapy-resistant cancers linked to chronic inflammation. Collaboration among specialists is essential to transform lab results into practical treatments. Larger patient populations and rigorous clinical studies are needed to verify safety and effectiveness. This research not only improves cancer treatment but also expands our knowledge of human–microbiota–parasite interactions, potentially benefiting autoimmune and inflammatory disease therapies. As research progresses, expect new paradigms in cancer treatment and personalized medicine development.

## Data Availability

No new data were created or analyzed in this study.

## Abbreviations

The following abbreviations are used in this manuscript:

5-ASA	5-amino salicylic acid
EGFR	Epidermal growth factor receptor
FexD	Fexaramine D
OCA	Obeticholic acid
T-βMCA	Tauro-β-muricholic acid
FXR	Farnesoid X receptor
M3R	Muscarinic acetylcholine receptor M3
LCA	Lithocholic acid
BAs	Bile acids
MFB	Metformin-butyrate
ROS	Reactive oxygen species
MAMPs	Microorganism-associated molecular patterns
TLRs	Toll-like receptors
TLRs	Through Toll-like receptors
EMT	Epithelial-mesenchymal transition
CRC	Colon cancer
LPS	Lipopolysaccharide
DCA	Deoxycholic acid
CSCs	Cancer stem-like cells
ERK	Extracellular regulated protein kinases
MAPK	Mitogen-activated protein kinase
PI3K	Phosphatidylinositol 3 kinase
AKT	Protein kinase B
VE-PTP	Vascular endothelial protein tyrosine phosphatase
AJs	Adhesion junctions
(VE)-cadherin	Vascular endothelial
TWIST1	Twist-related protein 1
EPHA2	Ephrin type-A receptor 2
VEGF	Vascular endothelial growth factor
MMPs	Matrix metalloproteinases
TGFB	Growth factor beta 1
FMO1	Flavin-containing monooxygenase
TMA	Trimethylamine
cutC/D	Choline TMA-lyase
CntA/B	L-carnitine oxygenase
SCFAs	Short-chain fatty acids
TMAO	Trimethylamine N-oxide
GM	Gut microbiota
